# A Comparative Study of Serum and Salivary Uric Acid Measurement in Pre-eclampsia and Normal Pregnancy in a Tertiary Care Hospital in Punjab: A Pilot Study

**DOI:** 10.7759/cureus.48457

**Published:** 2023-11-07

**Authors:** Isha Tapasvi, Parveen Rajora, Chaitanya Tapasvi, Amanpreet Sethi, Seema Grover

**Affiliations:** 1 Obstetrics and Gynecology, Guru Gobind Singh Medical College and Hospital, Faridkot, IND; 2 Radiodiagnosis, Guru Gobind Singh Medical College and Hospital, Faridkot, IND; 3 Pediatrics, Guru Gobind Singh Medical College and Hospital, Faridkot, IND

**Keywords:** high-risk pregnancy, pregnancy, salivary uric acid, serum uric acid, pre-eclampsia

## Abstract

Background

Pre-eclampsia is a multisystem progressive disorder associated with significant maternal and neonatal morbidity and mortality. It is essential to identify the potential indicators of associated complications in pre-eclampsia to improve pregnancy outcomes. Serum uric acid (UA) levels are increased in pregnancies complicated by pre-eclampsia. This study was performed to validate salivary UA as an alternative non-invasive biomarker to serum UA in pre-eclampsia.

Methodology

A total of 150 pregnant women were enrolled in the study. They were divided equally into three groups with 50 participants in each group. Group 1 included healthy normotensive pregnant women as control, group 2 included participants with non-severe pre-eclampsia and group 3 included participants with severe pre-eclampsia. Both salivary and serum UA were estimated in all the study participants and comparative analyses were done.

Results

Serum UA was elevated in 33(66%) and 48(96%) of participants in groups 2 and 3 respectively while saliva uric acid in 30(60%) and 43(86%) as compared with healthy controls who had serum UA raised in 14(28%) and salivary UA in 12(24%) with a significant p-value of 0.0001. The mean values of serum and salivary UA in group 1 were 4.5 ±1.16 mg/dl and 4.11±1.74 mg/dl respectively whereas in group 2 these were 6.12±1.86mg/dl and 5.96±1.90mg/dl and in group 3 these were 8.24±2.31mg/dl and 8.17±3.31mg/dl respectively. There was a significant correlation between serum and salivary UA values in groups 1 and 2, groups 1 and 3, and groups 2 and 3 with a p-value of 0. 001. The serum and salivary UA levels showed an increasing trend from healthy controls (group 1) to non-severe pre-eclampsia (group 2) with the highest values in severe pre-eclampsia (group 3). Oligohydramnios was present in 10(20%) cases in group 1 whereas 24(48%) cases in group 3. The appearance, pulse, grimace, activity, respiration (APGAR) score at 1 and 5 minutes was abnormal in 5(1.23%) and 7(1.33%) cases in group 1, 6(1.26%) and 7(1.29%) cases in group 2 and 5(1.73%) and 6(1.53%) cases in group 3. The area under curve (AUC) in group 2 was 0.778 for serum UA and 0.779 for salivary UA. In group 3, the AUC for serum UA values was 0.938 and for salivary UA 0.882. A statistically significant correlation between serum and salivary UA values in group 2 (non-severe pre-eclampsia) was found with a p-value of 0.001 and Pearson's correlation coefficient r of 0.738.

Conclusions

Serum and salivary UA levels correlate with the severity of pre-eclampsia with maximum levels in severe pre-eclampsia (group 3) followed by group 2 (non-severe pre-eclampsia) with the lowest levels in group 1 (healthy controls). The authors are of the opinion that a non-invasive salivary UA test can replace the serum UA test and can be a useful supplementation for conventional pre-eclampsia prediction.

## Introduction

Hypertensive disorders complicate 10% of pregnancies and pre-eclampsia (PE) about 3-5%. Various complications due to PE lead to almost 40,000 maternal deaths each year [1]. This disease has a multifactorial progressive spectrum which involves abnormal placentation and widespread maternal endothelial and vascular dysfunction leading to end-organ damage with clinical manifestations such as hypertension, proteinuria, systemic inflammation, and accumulation of various antiangiogenic factors. 

In normal pregnancies, there is an expansion of blood volume, which along with the increase in renal blood flow, glomerular filtration rate, and the uricosuric action of estrogen leads to a decrease in serum uric acid (UA) concentration. In pregnancies complicated by PE, there is a decrease in the UA excretion which results in oxidative stress and an increase in serum UA levels. The formation of reactive oxygen species in PE also contributes to hyperuricemia [2].

Limited access to advanced care in pregnancy particularly in developing countries can increase the risk of adverse maternal and fetal outcomes in PE. Early prediction of high-risk pregnancy cases by non-invasive diagnostic and screening tests can aid in favorable pregnancy outcomes. There is an utmost need to identify a safe, simple, effective, inexpensive, and reliable screening method that can detect impending complications in hypertensive disorders of pregnancy timely so that specific interventions can be planned accordingly.

Human saliva consists of 99% water with inorganic salts of sodium, potassium, calcium, chlorate, bicarbonate, phosphate, and organic compounds including UA, lactate, hormones, polypeptides, and proteins, such as immunoglobulins, enzymes, and mucins [3]. UA is the end product of the metabolic breakdown of purine nucleotides. The association of elevated UA levels during pregnancy has been examined as a potential indicator of risk for gestational hypertension, gestational diabetes mellitus, and related adverse birth outcomes. Different studies have concluded that monitoring of UA levels is essential in hypertensive disorders of pregnancy with both high and low maternal serum UA levels affecting the pregnancy outcome. The uses and benefits of UA detection in maternal saliva are that being non-invasive and cost-effective, salivary UA analysis can be used in population-based screening for various metabolic diseases in pregnancy [3]. Maternal saliva UA detection has the potential to aid in diagnostic research in various oxidative stress-related diseases in pregnancy [4-6]. 

There have been very few studies on levels of UA in PE in the northwestern region of Punjab, so there is a need for a non-invasive test that can help in screening high-risk cases of PE in early pregnancy. The purpose of this pilot study was to investigate the feasibility of salivary UA estimation as a non-invasive alternative to serum UA level in PE.

## Materials and methods

This descriptive cross-sectional study was done over a period of two years in a government tertiary care hospital in Punjab which included 150 pregnant women. The study received approval from the Institutional Ethics Committee of Guru Gobind Singh Medical College and Hospital, Faridkot, Punjab under reference number GGS/IEC/71. The participants were divided into 3 equal groups. Group 1 included healthy normotensive pregnant women as control (n=50). Group 2 included participants with non-severe pre-eclampsia (n=50). Group 3 included participants with severe pre-eclampsia (n=50). All women were enrolled in the study after informed consent as per the inclusion and exclusion criteria (Table 1). All the participants were subjected to detailed history and clinical examination. Relevant blood investigations were done as per the institutional protocol.

**Table 1:** Inclusion and exclusion criteria

Inclusion criteria	All antenatal women between 20 to 40 weeks of gestation who gave consent
Exclusion criteria	Participants with a history of liver disease, diabetes or hypertension, kidney disease, heart disease, or having twin/multiple pregnancies

Definitions

The study was based on criteria for the diagnosis of hypertension from the American College of Obstetricians and Gynecologists Task Force on Hypertension in Pregnancy. Non-severe pre-eclampsia was defined as BP ≥140/90 mmHg but <160/110 mmHg observed on at least 2 occasions at least 4 hours apart after 20 weeks of gestation in a previously normotensive patient with proteinuria ≥ 0.3g/24-hour, urine protein to creatinine ratio ≥0.3mg/dl, urine dipstick ≥1+ or serum transaminase concentration ≥ 2 times upper limit of normal range or platelets <100,000/µL or serum creatinine >1.1 mg/dL or pulmonary edema or liver transaminase levels at least twice the upper limit of normal or cerebral or visual symptoms. Severe pre-eclampsia was defined as BP of ≥160/110 mmHg observed on 2 occasions at least 4 hours apart while a patient is on bed rest with symptoms of central nervous system dysfunction, hepatic abnormality, serum transaminase concentration ≥2 times upper limit of the normal range, platelet count of < 100,000 platelets /µL, serum creatinine >1.1 mg/dL) or pulmonary edema. Proteinuria was defined as urinary protein excretion of ≥300 mg/24 hours. Pregnancy-induced hypertension was defined as BP ≥140/90 mmHg on two occasions at least 6 hours apart in women who were normotensive in the pre-pregnancy phase. Hyperuricemia was defined as elevated UA levels (≥4.5 mg/dL).

Collection and analysis of serum blood samples

2 ml of venous blood was collected after taking aseptic precautions in a sterile non-ethylenediaminetetraacetic acid (EDTA) vial. The samples were immediately sent for laboratory analysis. The blood sample was transferred into a sterile test tube and allowed to clot. Serum UA was done after centrifugation in a test tube.

Collection and analysis of saliva samples

The subjects were asked to rinse their mouth thoroughly and avoid drinking or eating at least 2 hours before collection of salivary samples. Unstimulated whole saliva (about 5 ml) was collected with the spitting method and sent to the laboratory for analysis. 

The sample was centrifuged, and UA estimation was done in the serum and saliva by enzymatic colorimetric method.

Principle of determination of serum and salivary uric acid levels

In the presence of uricase enzyme, UA is converted to allantoin (water-soluble metabolite) and hydrogen peroxide (H_2_O_2_) after which 4-amino-phenazone (phenol derivative) is converted to quinoid pigment in the presence of H_2_O_2_ and enzyme peroxidase. The intensity of red color dye is directly proportional to the concentration of UA and is determined photometrically.

## Results

A total of 150 antenatal women were enrolled equally in three groups with 50 participants in each group. The mean age for group 1, group 2, and 3 was 25.54, 24.78, and 26.10 years respectively. The groups showed no statistically significant difference in age and socioeconomic status of participants. The mean period of gestation was 37±2.4 weeks in group 1, 36±3.6 weeks in group 2, and 35±4.4 weeks in group 3. Thirty-two(64%) of primigravida patients in group 2 had non-severe pre-eclampsia which was statistically significant (p-value 0.004). Serum UA was elevated in 33(66%) and 48(96%) of participants in groups 2 and 3 respectively while saliva uric acid in 30(60%) and 43(86%), as compared with healthy controls who had serum UA raised in 14(28%) and salivary UA in 12(24%) with a significant p-value of 0.0001. Oligohydramnios was present in 10(20%) cases in group 1 whereas 24(48%) cases in group 3. Ultrasound evaluation of the participants revealed significant differences in Doppler parameters of healthy controls (group 1) and pre-eclampsia (groups 2 and 3) patients with a significant p-value of 0.001. Mode of delivery in terms of vaginal delivery and lower segment cesarean section had no significant correlation in any of the study groups. In neonatal outcome, respiratory distress was present in 3(6%) cases in group 1, 13(26%) in group 2, and 22(44%) cases in group 3 patients. The appearance, pulse, grimace, activity, respiration (APGAR) score at 1 and 5 minutes was abnormal in 5(1.23%) and 7(1.33%) cases in group 1, 6(1.26%) and 7(1.29%) cases in group 2, and 5(1.73%) and 6(1.53%) cases in group 3 respectively (Table 2).

**Table 2:** Clinico-demographic characteristics of study participants (N=150) POG: Period of gestation, IUGR: Intrauterine growth retardation, UA: Uric acid, MSL: Meconium-stained liquor, IUD: Intrauterine death, LSCS: Lower segment cesarean section, NVD: Normal vaginal delivery, PTVD: Preterm vaginal delivery, APGAR score: Appearance, pulse, grimace, activity, respiration score, NICU: Neonatal intensive care unit

Variables	Group						Chi-square value	p-value
	1		2		3			
Age	25.54	5.07	24.78	4.44	26.10	4.95	0.941	0.393
Socioeconomic status	19	38	20	40	26	52	2.335	0.311
POG	36.94	2.37	35.90	3.60	34.56	4.40	5.621	0.004
PARITY								
Primigravida	17	34%	32	64%	24	48%	15.276	0.004
Gravida 2	18	36%	14	28%	21	42%		
Gravida 3 and above	15	30%	4	8%	5	10%		
Serum UA > 5.1 mg/dl	14	28%	33	66%	48	96%	50.01	0.0001
Salivary UA > 5.1mg/dl	12	24%	30	60%	43	86%	39.475	0.0001
ULTRASOUND FINDING								
Oligohydramnios	10	20%	13	26%	24	48%	10.101	0.006
IUD	1	2%	2	4%	3	6%	1.042	0.594
Anhydramnios	2	4%	2	4%	2	4%	0.000	1.000
Polyhydramnios	2	4%	2	4%	3	6%	0.300	0.861
Doppler	7	14%	24	48%	25	50%	17.496	0.000
MODE OF DELIVERY								
Conservative	3	6%	1	2%	0	0%	17.688	0.126
Forceps delivery	0	0%	0	0%	1	2%		
LSCS	25	50%	29	58%	37	74%		
NVD	22	44%	18	36%	10	20%		
PTVD	0	0%	2	4%	2	4%		
NEONATAL OUTCOME								
Fetal/neonatal distress	3	6%	13	26%	22	44%	19.102	0.0001
IUGR	3	6%	6	12%	11	22%	5.54	0.059
MSL	8	16%	6	12%	7	14%	0.332	0.847
Alive	43	86%	41	82%	37	74%	2.394	0.302
Stillbirth	0	0%	1	2%	2	4%	2.041	0.360
Intrauterine death	1	2%	3	6%	3	6%	1.199	0.549
APGAR SCORE 1 MINUTE	5.78	1.23	5.69	1.26	5.04	1.73	3.607	0.030
APGAR SCORE 5 MINUTE	7.33	1.33	7.27	1.29	6.60	1.53	3.822	0.024
NICU admission	9	18%	20	40%	26	52%	7.3348	0.0255

Table 3 shows the mean values of serum and salivary UA in group 1 which were 4.5±1.16 mg/dl and 4.11±1.74 mg/dl respectively whereas in group 2 these were 6.12±1.86mg/dl and 5.96±1.90mg/dl and in group 3 these were 8.24±2.31 mg/dl and 8.17±3.31 mg/dl respectively. There was a significant correlation between serum and salivary UA values in groups 1 and 2, groups 1 and 3, and groups 2 and 3 with a p-value of 0. 001. The serum and salivary UA levels are showing an increasing trend from healthy controls (group 1) to mild pre-eclampsia (group 2) with the highest values in severe pre-eclampsia (group 3).

**Table 3:** Intergroup correlation UA: Uric acid

	Group 1		Group 2		Group 3		F	p-value	Group 1 vs 2	Group 1 vs 3	Group 2 vs 3
	Mean	SD	Mean	SD	Mean	SD			p-value	p-value	p-value
Serum UA mg/dl	4.50	1.16	6.12	1.86	8.24	2.31	52.069	0.000	0.000	0.000	0.000
Salivary UA mg/dl	4.11	1.74	5.96	1.90	8.17	3.31	34.577	0.000	0.000	0.000	0.000

**Figure 1:**
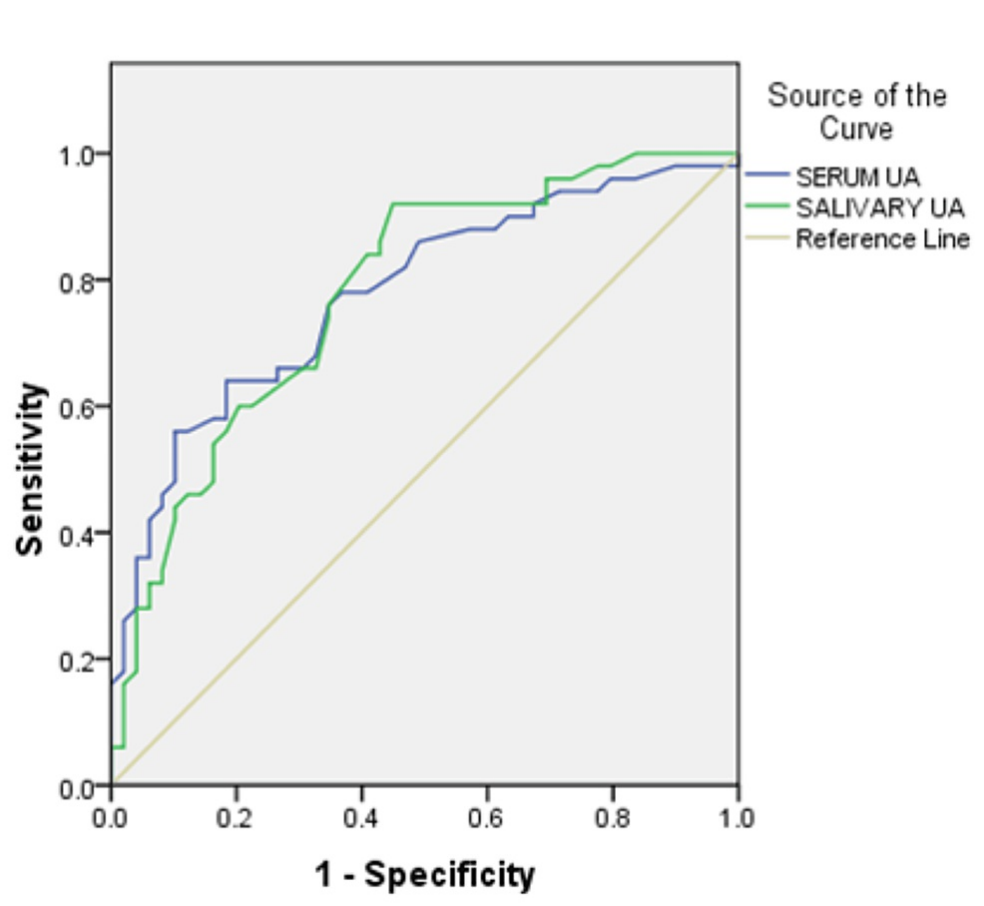
Receiver operating characteristic curve group 2 (non-severe pre-eclampsia) UA: Uric acid

**Figure 2:**
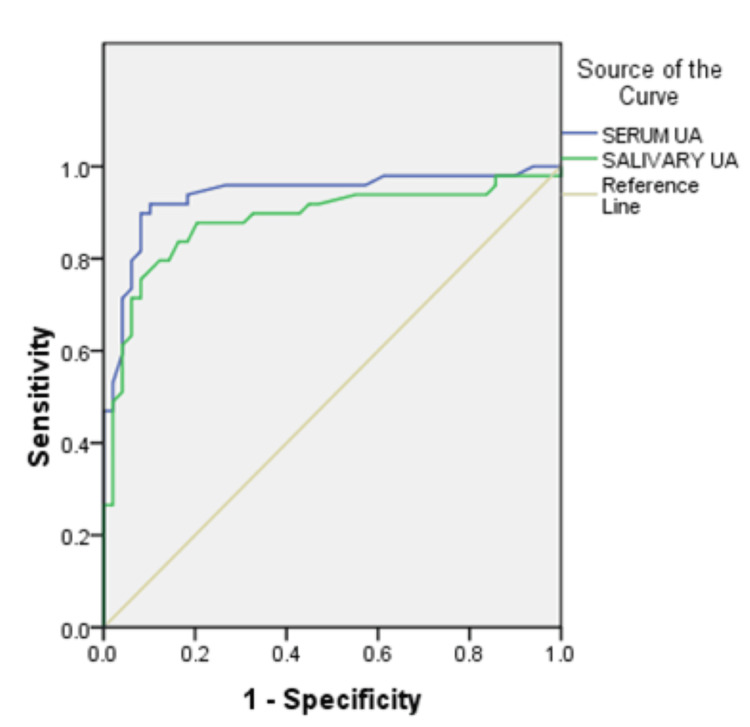
Receiver operating characteristic curve group 3 (severe pre-eclampsia) UA: Uric acid

The area under curve (AUC) in group 2 was 0.778 (78% accuracy) for serum UA and 0.779 for salivary UA. In group 3, AUC for serum and salivary UA values was 0.938 and 0.882 respectively which was more than that in group 2. The cut-off values of serum UA in group 2 after assessing the co-ordinates of curve were 4.75 mg/dl with a sensitivity of 76% and specificity of 65%. The cut-off values for salivary UA were 4.15 mg/dl with a 92% sensitivity and 55% specificity. In group 3, with serum UA, the cut-off values were 5.95 mg/dl with a 90% sensitivity and 92% specificity. Salivary UA in group 3 had cut-off values of 5.75 mg/dl with a sensitivity of 80% and 88% specificity. This suggests that increasing values of serum and salivary UA correlate with the severity of pre-eclampsia (Table 4; Figures 1, 2).

**Table 4:** Area under curve for serum and salivary UA UA: Uric acid

Test result variable(s)	Area under curve	Std. error	p-value	Asymptotic 95% confidence interval		Cut-off	Sensitivity	Specificity
				Lower bound	Upper bound			
Group 2								
Serum UA	0.778	0.047	0.001	0.687	0.869	4.65	78.0%	63.3%
Salivary UA	0.779	0.046	0.001	0.689	0.870	4.15	92.0%	55.1%
Group 3								
Serum UA	0.938	0.026	0.001	0.886	0.989	5.75	91.8%	89.8%
Salivary UA	0.882	0.037	0.001	0.809	0.955	5.15	87.8%	79.6%

The predictors of severe pre-eclampsia by multivariate regression analyses in group 3 were fetal/neonatal distress, period of gestation, serum uric acid, and salivary uric acid. Fetal/neonatal distress is the best predictor with a maximum odds ratio of 61.95 followed by serum UA, salivary UA, and period of gestation. In group 2, both non-reactive non-stress test (NST) and/or abnormal Doppler and serum UA are the predictors, with non-reactive NST and/or abnormal Doppler being a better predictor having an odds ratio of 3.58 (Table 5).

**Table 5:** Predictors for severe pre-eclampsia (multivariate analysis) POG: Period of gestation, UA: Uric acid, Primi: Primigravida, NST: Non-stress test, CI: Confidence interval

Variables	Group 2				Group 3			
	p-value	Odds ratio	95% CI for odds ratio		p-value	Odds ratio	95% CI for odds ratio	
			Lower	Upper			Lower	Upper
Parity reference primi	0.193				0.282			
Parity-1	0.373	0.593	0.187	1.875	0.150	0.154	0.012	1.964
Parity-2	0.076	0.264	0.060	1.151	0.189	0.134	0.007	2.694
Oligohydramnios					0.613	0.517	0.040	6.663
Abnormal doppler	0.040	3.583	1.059	12.119	0.092	11.169	0.675	184.684
Fetal / Neonatal Distress	0.157	3.148	0.643	15.420	0.012	61.955	2.432	1578.284
POG					0.044	0.718	0.520	0.991
Serum UA	0.011	1.722	1.135	2.612	0.001	4.992	1.885	13.215
Salivary UA	0.106	1.340	0.940	1.909	0.025	2.040	1.092	3.811

**Figure 3:**
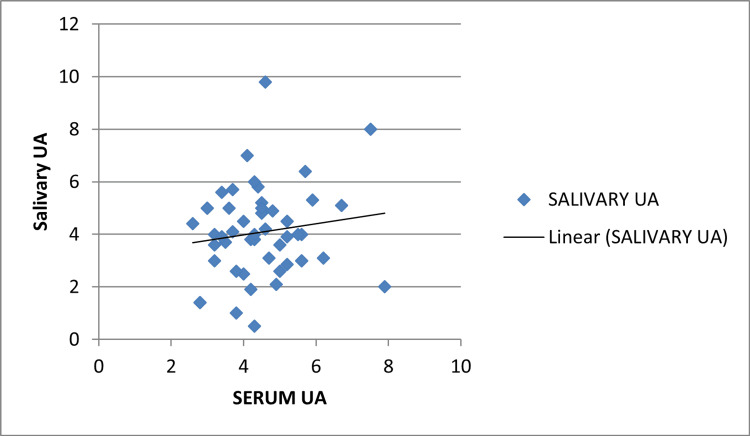
Pearson's correlation coefficient group 1 (control) UA: Uric acid

**Figure 4:**
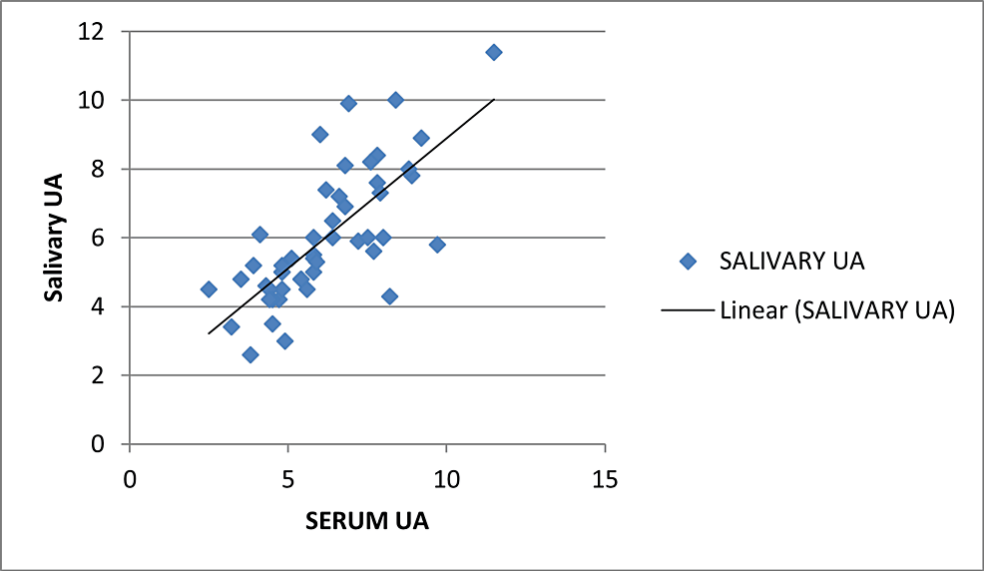
Pearson's correlation coefficient group 2 (non-severe pre-eclampsia) UA: Uric acid

**Figure 5:**
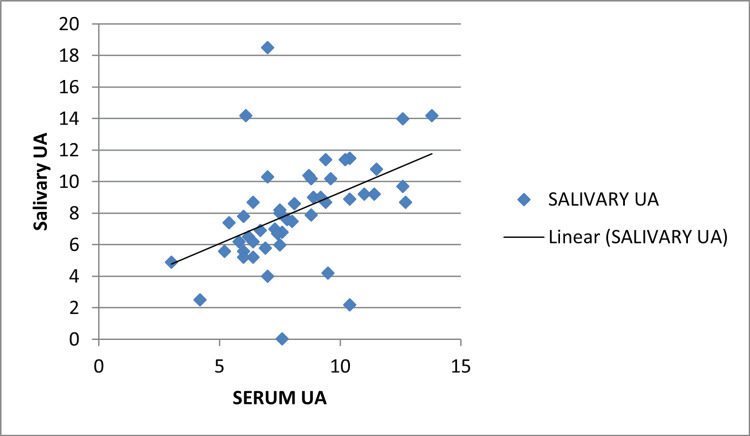
Pearson's correlation coefficient group 3 (severe pre-eclampsia) UA: Uric acid

A statistically significant correlation between serum and salivary UA with group 2 (mild pre-eclampsia) was found with a p-value of 0.001 and Pearson's correlation coefficient r=0.738. This correlation was not significant with group 1 with a p-value of 0.284, r=0.156. This correlation was also significant in group 3 with a p-value of 0.001 and r=0.456 but the r-value is less than with that of group 2 which shows that non-severe pre-eclampsia is more likely to be associated with a rise in serum and salivary UA (Figures 3, 4, 5).

## Discussion

Maternal serum uric acid (UA) studies in pregnancy primarily focus on high levels of UA, however, both low and high UA levels can be markers of oxidative stress, a biological state potentially linked to high maternal blood pressure recordings and impaired fetal growth [4].

In recent years, significant advances have been made toward the validation of salivary biomarkers for disease detection especially in various metabolic diseases. 

A nested case-control study has shown a significant positive correlation between UA and mean arterial pressure in the second trimester (r=+0.246, p=0.022) [6]. Our study has shown similar findings with significant correlation between group 2 (non-severe pre-eclampsia) and group 3 (severe pre-eclampsia) with serum and salivary UA levels. Thus, it can be inferred from our study that both serum and salivary UA levels are significantly raised in cases of pre-eclampsia.

Madaan et al. in their study have shown a significant association between the socioeconomic status of patients with serum and salivary UA levels whereas in our study there is no significant association between UA levels and socioeconomic status of participants [7]. Kim et al. in their study in 2018 investigated the relationship of socioeconomic status with pregnancy outcomes and concluded that the participants in the lower socio-economic group showed higher rates of abortion, cesarean delivery, pre-eclampsia, preterm delivery, and obstetrical hemorrhage as compared to those in the middle/high socioeconomic group [8]. The variation in results of these studies from our study related to the socioeconomic status of the participants may be attributed to the geographic variation in population along with the loco-regional availability and accessibility of the health care facilities to the population.

In a study of 58 hypertensive pregnant women by Paula et al., maternal diastolic blood pressure (BP) was higher in pregnant women with elevated UA levels, but systolic BP, gestational age, and birth weight were not significantly different [9]. The authors concluded that UA equal to or above 357 micromole/Lin in pregnant hypertensive women was associated with proteinuria and elevation of diastolic BP, but not with fetal outcome. In their study, Yuan et al. have reported the association of maternal UA with adverse birth outcomes and have suggested the utility of routine determination of maternal UA in late pregnancy [10].

In a study by Roberts et al., the authors concluded that higher serum UA levels in the second or third trimester increased the risk of adverse maternal and fetal outcomes [11]. Our study has also shown similar results of serum UA levels in relation to adverse maternal and fetal outcomes.

Deepashree et al. in their longitudinal study have described a significant positive correlation between UA levels in serum and saliva [12]. The authors have also suggested that as salivary UA reflects the changes in serum UA levels, it could reliably be used as an alternative to serum UA. Our study has also shown a positive correlation between serum and salivary UA levels with Pearson's correlation coefficient(r)of 0.738.

In a cross-sectional study by Singh et al., it was concluded that salivary UA levels have a linear correlation with serum UA levels [13]. Saliva UA (mean ± SD value 4.86 ± 2.37 mg/dl) of pregnant females with pre-eclampsia was significantly higher than that of healthy non-pregnant females (mean ± SD value 2.09 ± 1.33 mg/dl) with a significant p-value of p < 0.001. The authors have also compared serum UA of pregnant females with pre-eclampsia (mean ± SD value 6.63+2.78 mg/dl) with non-pregnant females (mean ± SD value 2.94+1.94 mg/dl) with a significant p-value of p<0.001. Kumar et al. in their study have found the mean ± SD values of 5.47±1.93 mg/dl for serum UA in women with gestational hypertension; 6.72±2.15 mg/dl in pre-eclampsia and 8.71±2.97 mg/dl in the eclamptic group [14]. In our study, mean ± SD values of serum and salivary UA in group 1 were 4.5±1.16 mg/dl and 4.11±1.74 mg/dl respectively. In group 2, these values were 6.12±1.86 mg/dl and 5.96±1.90 mg/dl respectively, and 8.24±2.31 mg/dl and 8.17±3.31 mg/dl respectively. The results of our study correlate with the findings of these studies as there is a rise in serum and salivary UA levels with increasing severity of pre-eclampsia. 

Riis et al. examined the validity of salivary UA as a non-invasive measure of serum UA and found a robust positive saliva and serum correlation for UA levels [15]. Gautam et al. in a descriptive cross-sectional study of 100 participants observed a linear correlation between serum and salivary values of UA (r = 0.334, p = 0.006) [16]. Our study also showed a linear correlation between serum and salivary uric acid (r=0.738, p-value 0.001).

In our study, there was no significant difference in the mode of delivery (vaginal /cesarean section) among the three groups with a p-value of 0.126. There was a discrepancy in the results of our study and the study by Madaan et al. in which the authors concluded that serum UA can predict the mode of delivery (normal vaginal or cesarean) among cases of severe pre-eclampsia [7]. This difference in results can be due to the variation in the spectrum of severity of pre-eclampsia in the study participants and the management protocols of the Institutes.

In our study, the increasing uric acid levels were associated with the severity of pre-eclampsia and the resultant admission of babies in NICU (p-value 0.0001). These results were similar to the study by Madan et al. and Püschl et al. [7,17]. In a recent study by Corominas et al. to assess the role of uric acid as a predictor of pre-eclampsia, it was found that its evaluation in the second and third trimesters would still provide useful information to timely referring a woman at risk to a more complex center [2].

In our study, multivariate regression analyses in group 3 showed that fetal distress, period of gestation, serum, and salivary UA were all predictors of severe pre-eclampsia with fetal distress being the best predictor (maximum odds ratio of 61.95). In group 2, Doppler ultrasound and serum UA were the predictors with Doppler ultrasound being the best predictor having an odds ratio of 3.58 and serum UA of 1.72. 

The increase in UA in early pregnancy precedes the reduction of glomerular filtration and hypovolemia associated with pre-eclampsia. The fetus, placenta, and maternal tissue are considered potential sources of UA in pregnant women. The increase in UA occurs as early as 10 weeks of pregnancy [18]. UA is a potential diagnostic tool in screening in the second and third trimesters and salivary UA can be used as a surrogate for serum UA screening.

Limitations of the study

This pilot study was done on a small sample size in a tertiary care government hospital in the northwestern region of the state of Punjab. Due to locoregional, socio-economic variations in population with limited availability and accessibility to healthcare facilities in this region, authors recommend multi-institutional studies to further validate the results of this study.

## Conclusions

In this study, the authors observed a significantly higher concentration of serum and salivary uric acid (UA) levels in pre-eclampsia (PE) as compared with normotensive pregnant women. A linear relationship was observed between salivary and serum UA levels. 

Serum and salivary UA levels correlate with the severity of PE with maximum levels in severe PE (group 3) followed by group 2 (non-severe PE) with the lowest levels in group 1 (healthy controls). The levels of serum and salivary UA were almost similar in all three groups. The authors are of the opinion that a non-invasive salivary UA test can replace the serum UA test and can be a useful supplementation for conventional PE prediction. This can make repeated sampling of UA in pregnancy easier and more patient-friendly with overall accuracy similar to serum UA levels. The authors recommend larger multi-institutional studies to further validate the findings of this study in a wider population.
